# The Economic Landscape of Global Rabies: A Scoping Review and Future Directions

**DOI:** 10.3390/tropicalmed10080222

**Published:** 2025-08-06

**Authors:** Molly Selleck, Peter Koppes, Colin Jareb, Steven Shwiff, Lirong Liu, Stephanie A. Shwiff

**Affiliations:** 1 Department of Economics, Colorado State University, Fort Collins, CO 80523, USA; peter.koppes@colostate.edu; 2 National Wildlife Research Center, Wildlife Services, Animal and Plant Health Inspection Services, U.S. Department of Agriculture, Fort Collins, CO 80521, USA; colin.jareb@usda.gov (C.J.); stephanie.a.shwiff@usda.gov (S.A.S.); 3 Department of Economics and Business, East Texas A&M University–Commerce, Commerce, TX 75428, USA; steven.shwiff@tamuc.edu (S.S.); lirong.liu@tamuc.edu (L.L.)

**Keywords:** rabies, economics, cost analysis, scoping review

## Abstract

Rabies remains a significant global public health concern, causing an estimated 59,000–69,000 human fatalities annually. Despite being entirely preventable through vaccination, rabies continues to impose substantial economic burdens worldwide. This study presents a scoping review of the economic research on rabies to determine overlaps and gaps in knowledge and inform future research strategies. We selected 150 studies (1973–2024) to analyze. The review categorizes the literature based on geographic distribution, species focus, and type of study. Findings indicate that economic studies are disproportionately concentrated in developed countries, such as the United States and parts of Europe, where rabies risk is low, while high-risk regions, particularly in Africa and Asia, remain underrepresented. Most studies focus on dog-mediated rabies, reflecting its dominant role in human transmission, while fewer studies assess the economic impacts of wildlife and livestock-mediated rabies. Case studies and modeling approaches dominate the literature, whereas cost–benefit and cost–effectiveness analyses—critical for informing resource allocation—are limited. The review highlights the need for more economic evaluations in rabies-endemic regions, expanded research on non-dog reservoirs, and broader use of economic methods. Addressing these gaps will be crucial for optimizing rabies control and supporting global initiatives to eliminate dog-mediated rabies by 2030.

## 1. Introduction

Rabies, an acute viral encephalomyelitis caused by the rabies virus, primarily affects the nervous systems of mammals. As one of the oldest known diseases, rabies has been documented for over 4000 years and is characterized by one of the highest case fatality ratios among infectious diseases, approaching 100% once clinical symptoms manifest [1]. It is estimated that rabies results in approximately 59,000 to 69,000 global fatalities each year, underscoring its significance as a public health concern [2,3]. Notably, while rabies is fatal once symptoms appear, it is entirely preventable through effective vaccination strategies.

Globally, the burden of rabies varies, influenced by regional characteristics and local control measures. In developed countries where canine rabies has been largely eliminated, the virus persists primarily in wildlife reservoirs, including bats, foxes, raccoons, and skunks [4]. In contrast, domestic dogs serve as the principal reservoir in many developing nations, accounting for the majority of global human rabies cases [5,6]. The economic and social impacts of rabies in canids are profound, leading researchers to focus on this variant for data collection and analysis. Hampson et al. [2] estimated the cost of human life lost due to rabies, utilizing disability-adjusted life years (DALYs), resulting in a global economic burden of canine rabies estimated at USD 8.6 billion. Similarly, Anderson & Shwiff [3] conducted a Monte Carlo simulation to value human life in this context, estimating the annual global economic impact of canine rabies to be approximately USD 124.2 billion. The ranges for these estimates are quite broad, reflecting the uncertainty in the data and the challenges of estimating the burden of this neglected disease.

Effective strategies to combat dog-mediated rabies hinge on governmental priorities, resource availability, and public education. Various approaches have been implemented globally, including local dog population management, parenteral and oral vaccination campaigns, or combinations thereof [7,8]. The success of rabies control programs relies on collaboration across multiple disciplines [9]. In 2015, a global strategic plan was launched to eliminate dog-mediated rabies by 2030, supported by major organizations such as the WHO, the Food and Agriculture Organization (FAO), the World Organization for Animal Health (OIE), and the Global Alliance for Rabies Control (GARC) [10] (p. 125). Economic evaluations of these interventions are essential for developing cost-effective management strategies and achieving the goal of eliminating dog-mediated rabies.

Several literature reviews have addressed economic aspects of rabies, notably Meltzer and Rupprecht [11], who examined economic questions surrounding postexposure prophylaxis (PEP) in humans and rabies control programs in animals. Systematic reviews of the cost-effectiveness of various control programs have been conducted, often focusing on specific geographic areas such as Africa or the Americas [12,13,14]. Anothaisintawee et al. [15] provided a review concentrated on economic evaluation studies related to rabies mitigation programs.

Research on the economics of rabies has identified critical areas of concern, including the economic burden from healthcare expenses, lost productivity, and PEP costs [2]. Furthermore, the economic impact extends to livestock and wildlife, indirectly affecting agricultural sectors and tourism [11]. Public health costs, particularly those associated with vaccination initiatives for pets and wildlife, are significant. The effectiveness of interventions, such as mass dog vaccination campaigns, wildlife vaccination efforts, and public awareness initiatives, has also been a focus of study [7,8]. Despite these valuable insights, a comprehensive examination of the literature on the global economics of rabies remains lacking. A thorough review is essential to assess data variability, long-term economic impacts, comprehensive cost analyses, and regional differences in economic estimates.

This paper presents a scoping review of the economic impact and the economic evaluation of rabies on a global scale. This study contributes to the existing literature in three important ways: first, it categorizes economic studies by location, species, and type of analysis; second, it identifies overlaps in research efforts and gaps in knowledge; and third, it discusses the implications of these findings and suggests potential avenues for future research. The remainder of this manuscript is organized as follows: In the next section, we outline the methodological strategy employed to collect the articles considered in this analysis. We then describe the methods employed to categorize each article by certain factors, followed by the results of our analysis. Lastly, the Section 4 examines the broader implications for rabies management and policy.

## 2. Materials and Methods

**Figure 1:**
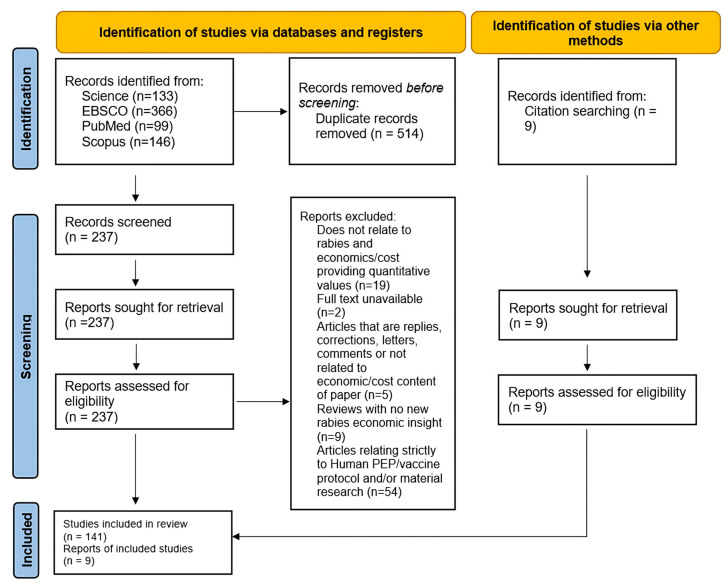
Flow diagram detailing the literature search and study selection process based on the PRISMA guidelines. This work is licensed under CC BY 4.0. To view a copy of this license, visit https://creativecommons.org/licenses/by/4.0/ (accessed on 24 July 2025).

We used a scoping review to identify the literature based on the methodological guidelines outlined by the Preferred Reporting Items for Systematic reviews and Meta-Analyses (PRISMA), with focus on the extension for Scoping Reviews (PRISMA-ScR) [16,17] (File S1). The literature search flow diagram for this scoping review is provided in Figure 1. The protocol of this scoping review has not been registered or published previously.

To capture the breadth of studies investigating the potential economic impact of rabies, the literature evaluating a myriad of costs associated with the disease was considered eligible and is reflected in the use of broad search terms. The scientific peer-reviewed literature from journals, edited book volumes, government reports, or other similar documents was considered eligible based on PRISMA-ScR guidance [17]. The following bibliographic databases were searched: Web of Science, EBSCO, PubMed, and Scopus. Primary search terms were looked for within the title, abstract, and keywords. The term “rabies” was paired with terms associated with economic impacts: economic(s), financial, cost, bioeconomic, and dollar(s). Publications published in any year were considered eligible, and the initial search yielded 744 articles. Details of the full electronic search strategy are outlined in Appendix A.

Publication titles and abstracts were screened and were excluded if they were a duplicate version of an already encountered publication (*n* = 230), did not report an economic cost of the disease (*n* = 19), were incomplete/unavailable text (*n* = 2), were in response to already encountered papers and were not relevant to economics (*n* = 5), or were a review with no novel economic findings (*n* = 9). Duplicates were screened and removed using Zotero software version 6.0.26 and then manually checked [18]. Additional articles were added manually from the references of the review articles included in the initial search (*n* = 9). The same primary search terms in the title, abstract, and index terms were considered as in the first stage. Once all relevant sources were identified and retrieved, each source was reviewed to ensure relevance. Based on these criteria, a total of 544 articles were excluded.

In the final eligibility stage, 204 articles were included. The next step was sorting these articles into categories—case studies, reviews, modeling, and letters. We removed any articles that were strictly related to human vaccination development or procedures (*n* = 54). The remaining 150 articles were compiled into a database (Table S1) to describe and summarize various characteristics of the publication. Using this database, we investigated the selected articles to summarize the research completed so far on the economics of rabies, specifically on variables such as location, species, institution, year, and methods.

## 3. Results

### 3.1. Timeline

**Figure 2:**
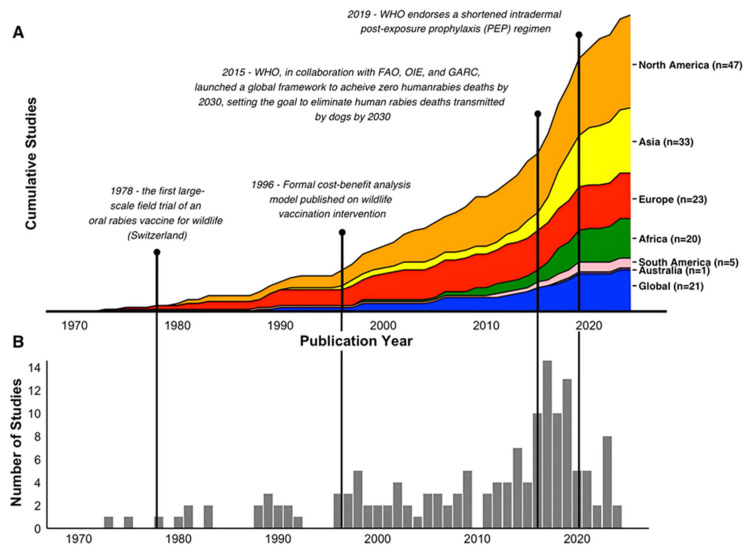
Temporal distribution and geographic focus of the reviewed literature. (**A**) shows the cumulative growth of studies across different regions, highlighting a significant increase in publications after key global initiatives (e.g., the 2015 WHO framework [10]). (**B**) presents the annual publication frequency, demonstrating a surge in research output in recent years.

For this analysis, the final database included 150 rabies-related economic studies published between 1973 and 2024 over the six continents with the presence of rabies (Figure 2). The number of studies published each year varies, with peaks in 2017 (15 studies) and 2019 (13 studies). The earliest study noted that met our review criteria was a case study in Argentina published in 1973 that included economic loss estimates in its summary of bat-transmitted rabies in the country [19]. The first formal cost–benefit analysis model was published by the Centers for Disease Control and Prevention (CDC) using North America-based data in 1996 [20]. The 2024 studies included research on trends in animal bites and rabies-related deaths in Northern Iran, along with an evaluation of the monetary costs, and a Canadian modeling study that assessed the efficiency of local rabies vaccination strategies for raccoons in an urban setting using individual-based modeling [21,22].

**Figure 3:**
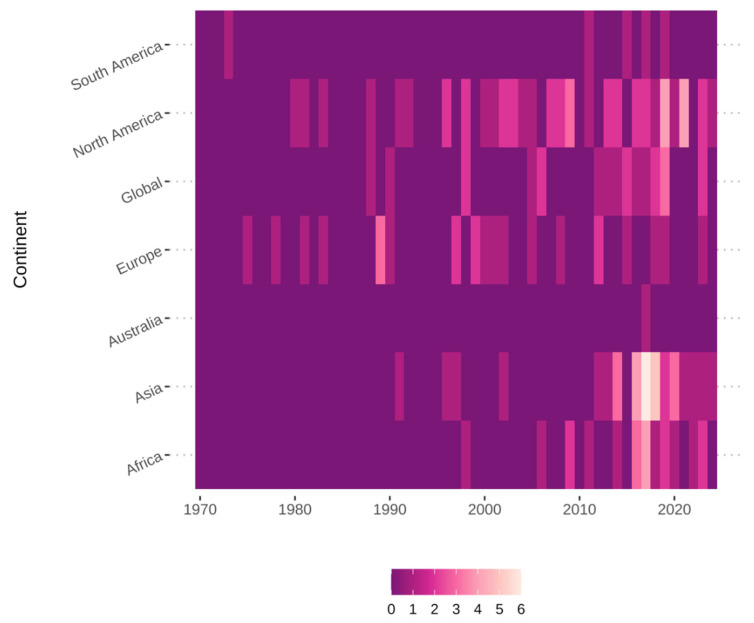
Annual publication counts by continent. The heatmap visually compares the research output across different continents over the study period, highlighting regions with consistently higher or lower publication numbers in specific years.

Four years, 2016–2019, stand out as notable publication years from the data, with 10, 15, 10, and 13 publications per year, respectively (Figure 2). Several interesting patterns emerge from these years, including that, in 2019, the total number of studies was driven up by the number of studies conducted in the USA (four studies) and globally (three studies). This cannot be said for the previous high publication years. In 2016 and 2017, each year had only one publication from the USA, while 2018 had none. In terms of global studies, 2017 and 2018 each had one publication, while 2016 had none. Examining the temporal changes by aggregating the countries to the continent or global study level indicates that studies conducted in Asia were mainly responsible for the spike from 2016 to 2019 (Figure 3).

### 3.2. Location and Research Institution

**Figure 4:**
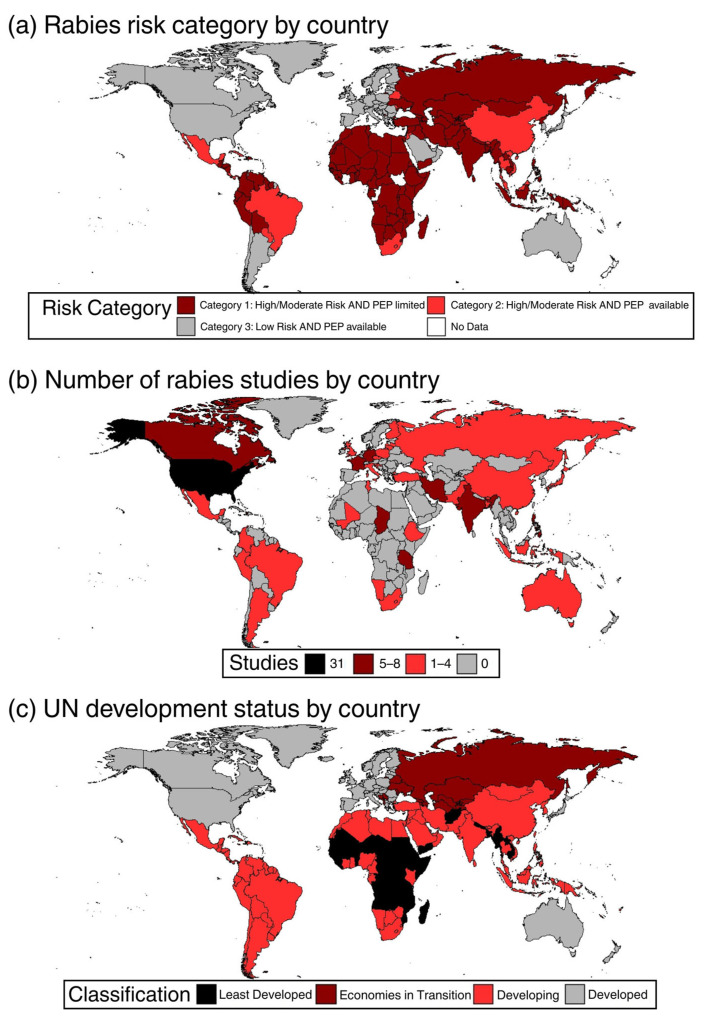
(**a**) Geographic distribution of rabies risk categories worldwide [23]; (**b**) the corresponding number of rabies-related studies identified in this review for each country; (**c**) the current economic classification of each country [24].

Over the study period, the database comprises rabies-related economic studies from 46 unique countries and 6 continents. The most frequently represented countries are the USA (31 studies), Haiti (8 studies), the Philippines (7 studies), Canada (7 studies), and India (6 studies) (Figure 4). A significant number of global or multi-country studies are also represented (18 studies). The first study included in this analysis was from South America (Argentina) in 1973 and focused on bat rabies (Figure 2). Europe was next with studies in Italy (1975) and Germany (1978). Economics-related studies from North America were first recorded in 1980 for the US and 1988 for Canada. The first global economic study was also conducted in 1988. Asia (Philippines) and Africa (Tunisia) conducted their first studies on the economics of rabies in 1991 and 1998, respectively (Figure 3).

Across the globe, 111 countries are considered at high risk for human rabies exposure [23]. Of those high-risk countries, only 19, or roughly 17%, had published studies on the economics of rabies. Of those 19 countries, 7 were in Africa, 6 in Asia, 3 in South America, and the final 3 were in the Middle East and the Caribbean. This result indicates that most of the high-risk areas in Africa and Asia have not examined the economics of rabies. Overlapping the 19 countries with rabies economic studies, which are also high risk for human rabies exposure, with basic country economic conditions, reveals that six of these countries fall into the category of “least developed countries” (Figure 4). The remaining fall into the category of “developing countries”.

**Figure 5:**
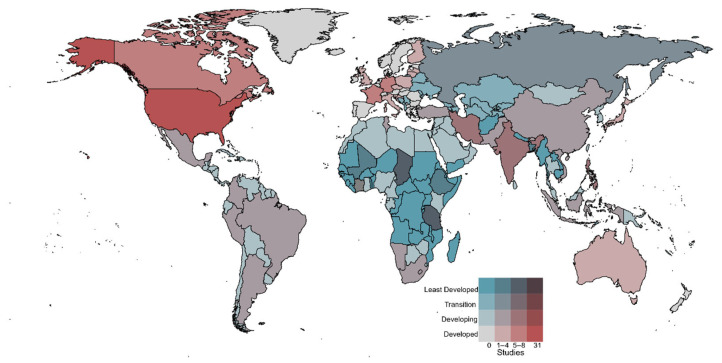
Geographic representation of the volume of rabies economic studies, overlaid with the development status of the corresponding countries, illustrated using a choropleth map.

Examining the number of studies from regions where rabies exposure risk levels for humans are moderate to low provides insight into the relationship between the potential for human rabies exposure and economic development. For example, the US is considered at low risk for human exposure to rabies, likely because of the presence of rabies in wildlife as opposed to dogs. However, it leads the world in the number of studies conducted on the economics of rabies. Canada, parts of Europe (France, Germany, Switzerland, Italy, the UK, the Netherlands, Finland, Slovenia, and Croatia), Argentina, and Australia have also conducted numerous studies. They, too, are considered at low risk for human exposure to rabies for the same reasons as the US and, for the most part, are considered “developed countries” (Figure 5).

The dataset includes papers affiliated with a diverse range of institutions. In total, publications from over 90 different institutions were identified. The most frequently represented institutions include the United States Department of Agriculture’s National Wildlife Research Center (23 papers) and the Centers for Disease Control and Prevention (15 papers). Beyond these top contributors, the remaining papers are evenly distributed among other institutions, suggesting a widespread interest across government agencies, public health organizations, and academic institutions both nationally and internationally. Funding sources, when explicitly stated, also came from a widespread range of sources. Notable contributors were the UBS Optimus Foundation (18 papers), the Wellcome Trust (14 papers), and the Global Alliance for Rabies Control (6 papers). These organizations, representing philanthropic, global health, and disease-specific initiatives respectively, highlight the multifaceted support driving rabies research.

### 3.3. Species

**Figure 6:**
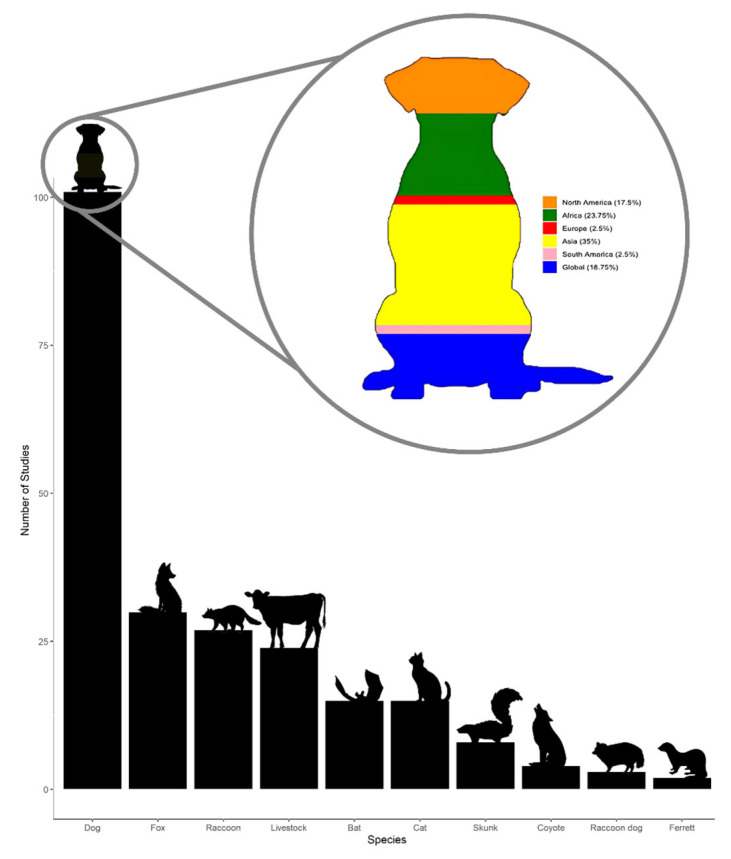
Species-specific focus of the reviewed literature. Dogs were the most frequently studied species, followed by foxes and raccoons. Continent breakdown of dog-focused studies provided in popout.

Much of the world is impacted by dog rabies, except in the cases of developed countries, where dog rabies has been eliminated, and wildlife has become the main reservoir for rabies [10]. Studies on the economics of dog-mediated rabies form the majority of those included in this analysis (Figure 6). For these studies, research was focused on canine vaccination programs, post-exposure prophylaxis (PEP), epidemiological surveillance, and economic assessments. Case studies document rabies outbreaks in dogs, particularly in endemic regions such as Africa, Asia, and Latin America. Modeling studies related to dog rabies assess transmission dynamics and vaccination strategies. When looking at specifically dog-focused studies, there was a noticeable difference in study location compared to all species, with more representation from Africa and Asia (Figure 6). Non-dog-related rabies studies examine wildlife reservoirs, such as bat rabies, and rabies in wild carnivores, such as foxes, raccoons, and skunks, and were primarily focused on North America and Europe. Livestock studies assess economic impacts and spillover risks in agricultural settings of rabies mediated by a variety of hosts to livestock, most especially cattle.

### 3.4. Type of Analysis

**Figure 7:**
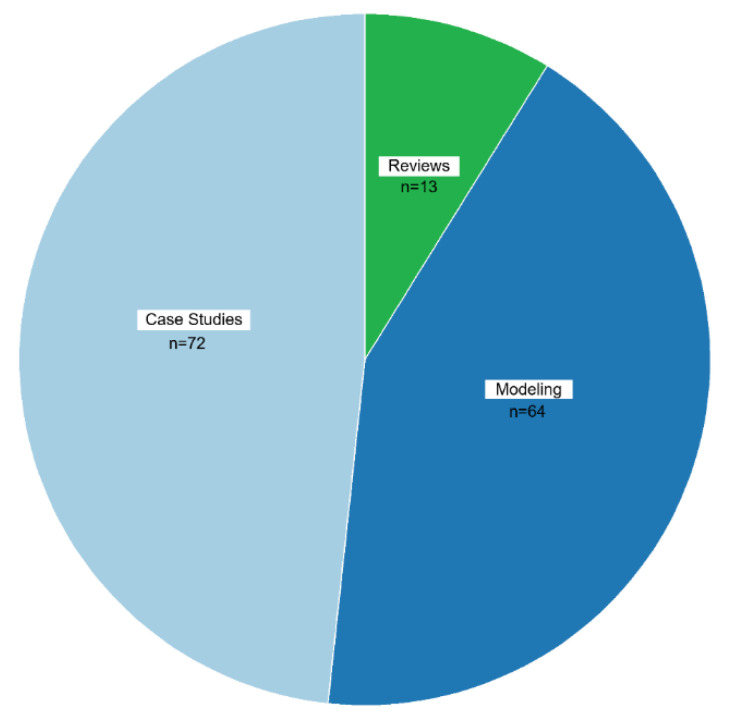
Distribution of economic rabies studies by study type.

The dataset is comprised of various study types, with the most common being case studies (72 studies). Case studies within the context of this review tended to focus on examining the economics of a particular rabies prevention or elimination campaign or human rabies exposure. Case studies were often limited by species and location, meaning that broad extrapolation of findings was limited. Modeling-based studies (64 studies), which include both prospective and retrospective approaches, were most often represented by studies that attempted to examine the hypothetically optimal level and mix of rabies control measures to determine the most cost-effective strategy. The dataset also contains systematic reviews (13 studies) (Figure 7). Cost–benefit analyses (CBAs) or Cost–effectiveness analysis (CEA) comprised 38 studies. A CBA typically involves comparing the monetary costs associated with management or elimination of the disease against the monetary benefits of preventing or avoiding the disease in humans, companion animals, wildlife, and livestock. Costs are typically measured by things such as vaccines, baits, and personnel, while benefits are measured as the prevention or cessation of PEP, PrEP, livestock losses, companion animal losses, medical treatment, and DALYs. CEA is a method used to evaluate the efficiency of healthcare interventions by comparing their costs with their health outcomes. It aims to determine which intervention provides the best value for money in terms of health benefits. For example, in the case of wildlife rabies prevention, a CEA might compare the costs of an oral rabies vaccine campaign vs. trap–vaccinate–release vs. culling to determine which method resulted in the largest reduction in PEP. A total of 61 studies did not incorporate any type of formal economic assessment.

## 4. Discussion

As the primary objective of this scoping review was to map the breadth and nature of the economic literature on rabies, our findings demonstrate that this body of evidence is imbalanced, showing a pronounced lack of economic studies from high-risk, endemic regions and an underutilization of comprehensive economic evaluation methods such as cost–benefit and cost–effectiveness analyses. While rabies remains a substantial public health and economic burden, particularly in regions where dog-mediated transmission persists, the distribution of research efforts does not align proportionally with areas of highest risk. The majority of economic studies originate from developed countries, such as the United States and parts of Europe, where the risk of human rabies exposure is relatively low due to well-established control measures. Conversely, many high-risk regions in Africa and Asia, where rabies poses the greatest human and economic threat, have produced comparatively few economic studies. This discrepancy underscores the need for increased research efforts in endemic regions to better inform cost-effective prevention and control strategies.

A notable pattern emerging from this review is the dominance of case studies and modeling-based analyses. Case studies, while valuable for assessing the effectiveness of specific interventions, are often geographically and temporally limited, making broad generalizations difficult. Modeling studies provide crucial insights into cost-effective strategies, yet their accuracy depends on the availability of high-quality data, which remains scarce in many high-risk regions. For example, rabies deaths are significantly underreported across the globe, likely due to inadequate reporting systems, limited access to healthcare in rural areas, and a lack of awareness about the disease [25]. Cost–benefit analyses and cost–effectiveness studies, though essential for guiding resource allocation, are underrepresented in the literature, particularly in countries with limited rabies control infrastructure. Expanding such studies in these areas could offer a clearer understanding of the economic feasibility of various intervention strategies, helping policymakers allocate resources more effectively.

Another key insight from this review is the strong focus on dog-mediated rabies, which reflects the global burden of the disease. However, economic research on non-dog reservoirs, such as wildlife, remains limited despite their relevance in rabies transmission and control. In high-income countries where canine rabies has been largely eliminated, wildlife species such as bats, raccoons, and foxes serve as primary reservoirs, influencing public health costs, livestock losses, and rabies prevention efforts. Future research should explore the economic implications of wildlife-mediated rabies and the cost-effectiveness of control strategies, such as oral vaccination programs.

The observed temporal trends in publication frequency also highlight shifting research priorities. Peaks in publication output during specific years suggest that external factors, such as international funding initiatives, disease outbreaks, or policy changes, influence the research agenda. However, the lack of consistent growth in rabies economic studies indicates that sustained investment in economic research remains a challenge. Given the ongoing global push to eliminate dog-mediated rabies by 2030, continued economic evaluation of intervention strategies is essential to assess progress and adapt policies accordingly.

This scoping review primarily focused on mapping the broad characteristics and gaps in the rabies-related economic literature, highlighting the diverse methodologies employed within these studies. Economic evaluations of rabies captured in this review utilize various modeling techniques, ranging from simple cost calculations to more sophisticated decision-analytic models (e.g., SEIR (susceptible–exposed–infectious–removed) models, decision trees) that simulate disease progression and intervention impacts over time. The choice of measured health outcomes, such as DALYs (disability-adjusted life years), QALYs (quality-adjusted life years), or simply averted cases/deaths, significantly influences the interpretation and comparability of economic findings [15]. Ultimately, these models are helpful for estimating long-term costs and benefits of potential medical interventions, revealing potential paths forward towards elimination of this zoonosis [15]. The elimination of any zoonosis is extremely complex, requiring thorough and thoughtful examination of all the tools available, especially those tools that are economically efficient [9]. While economics cannot capture all of the potential benefits and costs of zoonotic interventions, it does provide a standardized methodology by which interventions can be compared, allowing for an optimal utilization of limited resources in the fight against zoonosis.

Several recommendations for further research can be identified from this review and grouped into general categories, including economic studies in high-risk endemic regions, economic evaluation of non-dog rabies reservoirs, and methodological advancements in rabies economic research. This review identified the need for targeted CBA or CEA analyses, longitudinal economic impact studies, assessments of current reporting systems, and economic evaluation of integrated control programs, especially in high-risk endemic regions. One such suggestion may be to examine the benefits and costs of a more judicious use of PrEP to prevent human rabies deaths. In terms of potential economic evaluations of non-dog rabies reservoirs, particular emphasis should be focused on a CEA of wildlife rabies control, estimating the economic impact of wildlife-mediated rabies on specific sectors and modeling the economic consequences of spillover events. A suggestion for a study for this group may be to examine successful raccoon rabies elimination programs in Canada or the US. Lastly, to make methodological advancements in rabies economic research, future studies should promote the development of a standardized economic evaluation framework, refine data collection methods, and integrate non-monetary benefits and costs. Futures studies should focus on creating and validating standardized frameworks and guidelines for conducting economic evaluations of rabies interventions, particularly tailored for low-resource settings, to improve comparability across studies.

In conclusion, while substantial progress has been made in understanding the economics of rabies, critical gaps remain in geographic coverage, study diversity, and economic assessment methodologies. Addressing these gaps through targeted research in high-risk regions, increased focus on non-dog reservoirs, and broader application of cost–benefit and cost–effectiveness analyses will be essential for optimizing global rabies control efforts. Strengthening interdisciplinary collaborations and enhancing data collection efforts will further support evidence-based decision-making, ultimately contributing to the success of global rabies elimination initiatives.

## Data Availability

The original contributions presented in this study are included in the article/Supplementary Materials. Further inquiries can be directed to the corresponding author.

## Supplementary Materials

The following supporting information can be downloaded at https://www.mdpi.com/article/10.3390/tropicalmed10080222/s1: Table S1: Global Rabies Economics Scoping Review—Master Database 2025 [2,3,5,11,14,15,20,21,22,26,27,28,29,30,31,32,33,34,35,36,37,38,39,40,41,42,43,44,45,46,47,48,49,50,51,52,53,54,55,56,57,58,59,60,61,62,63,64,65,66,67,68,69,70,71,72,73,74,75,76,77,78,79,80,81,82,83,84,85,86,87,88,89,90,91,92,93,94,95,96,97,98,99,100,101,102,103,104,105,106,107,108,109,110,111,112,113,114,115,116,117,118,119,120,121,122,123,124,125,126,127,128,129,130,131,132,133,134,135,136,137,138,139,140,141,142,143,144,145,146,147,148,149,150,151,152,153,154,155,156,157,158,159,160,161,162,163,164,165,166]; File S1: PRISMA-ScR-Checklist_The Economic Landscape of Global Rabies.

## Abbreviations

The following abbreviations are used in this manuscript:

DALYs	Disability-adjusted life years
WHO	Directory of open access journals
FAO	Food and Agriculture Organization
OIE	World Organization for Animal Health
GARC	Global Alliance for Rabies Control
PEP	Postexposure prophylaxis
PRISMA	Preferred Reporting Items for Systematic Reviews and Meta-Analyses
ScR	Scoping Review
CDC	Centers for Disease Control and Prevention
CBA	Cost–benefit analyses
CEA	Cost–effectiveness analysis

## Appendix A

**Table A1:** Electronic search strategy for the Scoping review (conducted 1 October 2024).

Database	Query	# of Results
Web of Science	TI = Rabies AND TI = Economic OR Financial OR Cost OR Bioeconomic	115
	TI = Rabies AND AB = Dollar	18
EBSCO Host	TI = Rabies AND TI = Economic OR Financial OR Cost OR Bioeconomic	324
	TI = Rabies AND AB = Dollar	42
PubMed	TI = Rabies AND TI = Economic OR Financial OR Cost OR Bioeconomic	97
	TI = Rabies AND TIorAB = Dollar	2
Scopus	TI = Rabies AND TI = Economic OR Financial OR Cost OR Bioeconomic	125
	TI = Rabies AND AB/TI/KEYW = Dollar	21
	Total:	744

## References

[1] Tarantola A. (2017). Four thousand years of concepts relating to rabies in animals and humans, its prevention and its cure. Trop. Med. Infect. Dis..

[2] Hampson K., Coudeville L., Lembo T., Sambo M., Kieffer A., Attlan M., Barrat J., Blanton J.D., Briggs D.J., Cleaveland S. (2015). Estimating the global burden of endemic canine rabies. PLoS Neglected Trop. Dis..

[3] Anderson A., Shwiff S.A. (2015). The cost of canine rabies on four continents. Transbound. Emerg. Dis..

[4] Ma X., Monroe B.P., Cleaton J.M., Orciari L.A., Li Y., Kirby J.D., Blanton J.D. (2018). Rabies Surveillance in the United States During 2017. J. Am. Vet. Med. Assoc..

[5] Shwiff S., Hampson K., Anderson A. (2013). Potential economic benefits of eliminating canine rabies. Antivir. Res..

[6] World Health Organization (2013). WHO Expert Consultation on Rabies: Second Report.

[7] Elmore S.A., Chipman R.B., Slate D., Huyvaert K.P., VerCauteren K.C., Gilbert A.T. (2017). Management and modeling approaches for controlling raccoon rabies: The road to elimination. PLoS Neglected Trop. Dis..

[8] Tiwari H.K., Gogoi-Tiwari J., Robertson I.D. (2021). Eliminating dog-mediated rabies: Challenges and strategies. Anim. Dis..

[9] VerCauteren K.C., Ellis C., Chipman R., DeLiberto T., Shwiff S., Slate D., Frey S.N. Rabies in North America: A model of the one health approach. Proceedings of the 14th Wildlife Damage Management Conference.

[10] World Health Organization (2018). WHO Expert Consultation on Rabies: Third report.

[11] Meltzer M.I., Rupprecht C.E. (1998). A review of the economics of the prevention and control of rabies: Part 2: Rabies in dogs, livestock and wildlife. Pharmacoeconomics.

[12] Jibat T., Hogeveen H., Mourits M.C.M. (2015). Review on dog rabies vaccination coverage in Africa: A question of dog accessibility or cost recovery?. PLoS Neglected Trop. Dis..

[13] Shwiff S.A., Anderson A., Nadin-Davis S., Rupprecht C.E., Dykes N.L., Childs J.E. (2023). The health economics of rabies in the Americas: An historical summary and a synthesis of the literature. History of Rabies in The Americas: From The Pre-Columbian to The Present.

[14] Tunas I.K., Putra M.P.H., Sutiasa I.M., Budayanti N.N.S., Antara I.N.L.W., Sudhana I.B.P. (2023). Cost-effectiveness analysis of rabies control program, 2008–2017: A systematic review. J. Health Manag..

[15] Anothaisintawee T., Genuino A.J., Thavorncharoensap M., Youngkong S., Rattanavipapong W., Meeyai A., Chaikledkaew U. (2019). Cost-effectiveness modelling studies of all preventive measures against rabies: A systematic review. Vaccine.

[16] Page M.J., McKenzie J.E., Bossuyt P.M., Boutron I., Hoffmann T.C., Mulrow C.D., Shamseer L., Tetzlaff J.M., Akl E.A., Brennan S.E. (2021). The PRISMA 2020 Statement: An Updated Guideline for Reporting Systematic Reviews. BMJ.

[17] Tricco A.C., Lillie E., Zarin W., O’Brien K.K., Colquhoun H., Levac D., Moher D., Peters M.D.J., Horsley T., Weeks L. (2018). PRISMA Extension for Scoping Reviews (PRISMA-ScR): Checklist and Explanation. Ann. Intern. Med..

[18] (2023). Zotero.

[19] de Diego A.I., Valotta J.R. Bat-Transmitted Rabies. Situation in Argentina; Sociedad de Medicina Veterinaria, Buenos Aires, Argentina, 1973; pp. 275–295.

[20] Noah D.L., Smith M.G., Gotthardt J.C., Krebs J.W., Green D., Childs J.E. (1996). Mass human exposure to rabies in New Hampshire: Exposures, treatment, and cost. Am. J. Public Health.

[21] Kiakalayeh A.D., Gharib Z., Mohammadi R., Kanafi Vahed L., Davoudi-Kiakalayeh S. (2024). Trends in animal bites and rabies-related deaths in northern Iran: Implications for public health interventions. Arch. Iran. Med..

[22] Bastille-Rousseau G., Gorman N.T., McClure K.M., Nituch L., Buchanan T., Chipman R.B., Gilbert A.T., Pepin K.M. (2024). Assessing the efficiency of local rabies vaccination strategies for raccoons (Procyon lotor) in an urban setting. J. Wildl. Dis..

[23] Henry R.E., Blanton J.D., Angelo K.M., Pieracci E.G., Stauffer K., Jentes E.S., Rao S.R., Turabelidze G., White P., Blazes D.L. (2022). A country classification system to inform rabies prevention guidelines and regulations. J. Travel Med..

[24] United Nations Department of Economic and Social Affairs (2025). World Economic Situation and Prospects 2025. https://www.un.org/development/desa/dpad/publication/world-economic-situation-and-prospects-2025/.

[25] Taylor L.H., Hampson K., Fahrion A., Abela-Ridder B., Nel L.H. (2017). Difficulties in estimating the human burden of canine rabies. Acta Trop..

[26] Abbas S.S., Kakkar M., Rogawski E.T. (2014). Costs Analysis of a Population Level Rabies Control Programme in Tamil Nadu, India. PLoS Neglected Trop. Dis..

[27] Abbasi M., Barfar E., Hazratian T., Abbasi R. (2018). Estimating the Cost of Prevention and Control of Rabies: A Case Study in the Northwest of Iran. Evid. Based Health Policy Manag. Econ..

[28] Aicha E. (2016). Economic Impact of Animal and Human Rabies Prevention and Control in Tunisia between 2012 and 2016. Int. J. Infect. Dis..

[29] Amparo A.C.B., Jayme S.I., Roces M.C.R., Quizon M.C.L., Villalon E.E.S., Quiambao B.P., Baquilod M.S., Hernandez L.M., Taylor L.H., Nel L.H. (2018). The Evaluation of Operating Animal Bite Treatment Centers in the Philippines from a Health Provider Perspective. PLoS ONE.

[30] Anderson A., Kotzé J., Shwiff S.A., Hatch B., Slootmaker C., Conan A., Knobel D., Nel L.H. (2019). A Bioeconomic Model for the Optimization of Local Canine Rabies Control. PLoS Neglected Trop. Dis..

[31] Anderson A., Shwiff S., Gebhardt K., Ramírez A.J., Shwiff S., Kohler D., Lecuona L. (2014). Economic Evaluation of Vampire Bat (*Desmodus Rotundus*) Rabies Prevention in Mexico. Transbound. Emerg. Dis..

[32] Anderson A., Shwiff S.S., Shwiff S.A. (2014). Economic Impact of the Potential Spread of Vampire Bats into South Texas. Proc. Vertebr. Pest Conf..

[33] Anyiam F., Lechenne M., Mindekem R., Oussigéré A., Naissengar S., Alfaroukh I.O., Mbilo C., Moto D.D., Coleman P.G., Probst-Hensch N. (2017). Cost-Estimate and Proposal for a Development Impact Bond for Canine Rabies Elimination by Mass Vaccination in Chad. Acta Trop..

[34] Asokkumar M., Ganesan P.I., Sekar M., Anuradha P., Balakrishnan S. (2016). Vaccination Studies against Rabies in Farm and Pet Animals Using Different Immunization Routes. Indian Vet. J..

[35] Aubert M.F.A. (1999). Costs and Benefits of Rabies Control in Wildlife in France. OIE Rev. Sci. Et Tech..

[36] Babazadeh T., Nikbakhat H.A., Daemi A., Yegane-Kasgari M., Ghaffari-Fam S., Banaye-Jeddi M. (2016). Epidemiology of Acute Animal Bite and the Direct Cost of Rabies Vaccination. J. Acute Dis..

[37] Batan I.W., Lestyorini Y., Milfa S., Iffandi C., Nasution A.A., Faiziah N., Rasdiyanah, Herbert, Palgunadi N.W.L., Suatha I.K. (2014). Economic losses of rabies in Bali. J. Vet..

[38] Bätza H.J., Fooks A.R., Müller T. (2012). Cost of National Wildlife Rabies Elimination Programmes.

[39] Bedeković T., Šimić I., Krešić N., Lojkić I. (2019). Influence of Different Factors on the Costs and Benefits of Oral Vaccination of Foxes against Rabies. Zoonoses Public Health.

[40] Bellani L., Gagliardi G., Mantovani A., Morganti L., Prosperi S., Sanguinetti V. (1975). Epidemiology, socio-economic importance and control of rabies in Italy. Vet. Ital..

[41] Benavides J.A., Rojas Paniagua E., Hampson K., Valderrama W., Streicker D.G. (2017). Quantifying the Burden of Vampire Bat Rabies in Peruvian Livestock. PLoS Neglected Trop. Dis..

[42] Bennett R.M. (1997). Non-Market Costs of Rabies Policy. Vet. Rec. J. Br. Vet. Assoc..

[43] Beyene T.J., Fitzpatrick M.C., Galvani A.P., Mourits M.C.M., Revie C.W., Cernicchiaro N., Sanderson M.W., Hogeveen H. (2019). Impact of One-Health Framework on Vaccination Cost-Effectiveness: A Case Study of Rabies in Ethiopia. One Health.

[44] Birhane M.G., Miranda M.E.G., Dyer J.L., Blanton J.D., Recuenco S. (2016). Willingness to Pay for Dog Rabies Vaccine and Registration in Ilocos Norte, Philippines (2012). PLoS Neglected Trop. Dis..

[45] Bogel K., Meslin F.-X. (1990). Economics of Human and Canine Rabies Elimination: Guidelines for Programme Orientation. Bull. World Health Organ..

[46] Borse R.H., Atkins C.Y., Gambhir M., Undurraga E.A., Blanton J.D., Kahn E.B., Dyer J.L., Rupprecht C.E., Meltzer M.I. (2018). Cost-Effectiveness of Dog Rabies Vaccination Programs in East Africa. PLoS Neglected Trop. Dis..

[47] Bucher A., Dimov A., Fink G., Chitnis N., Bonfoh B., Zinsstag J. (2023). Benefit-Cost Analysis of Coordinated Strategies for Control of Rabies in Africa. Nat. Commun..

[48] (1989). Bulletin Épidémiologique Hebdomadaire Vulpine Rabies: Cost-Benefit Analysis of the Medical Prophylaxis of Vulpine Rabies. Bull. Épidémiologique Hebd. https://www.santepubliquefrance.fr/revues/beh/bulletin-epidemiologique-hebdomadaire.

[49] Castillo-Rodríguez L., Castañeda-Orjuela C.A., De La Hoz-Restrepo F. (2015). Cost-Effectiveness Analysis of Vaccination against Rabies in Dogs in Colombia. Value Health.

[50] Chang H.-G.H., Eidson M., Noonan-Toly C., Trimarchi C.V., Rudd R., Wallace B.J., Smith P.F., Morse D.L. (2002). Public Health Impact of Reemergence of Rabies, New York. Emerg. Infect. Dis..

[51] Chen Q., Liu Q., Gong C., Yin W., Mu D., Li Y., Ding S., Liu Y., Yang H., Zhou S. (2023). Strategies to inTerrupt RAbies Transmission for the Elimination Goal by 2030 In China (STRATEGIC): A Modelling Study. BMC Med..

[52] Chipman R.B., Cozzens T.W., Shwiff S.A., Biswas R., Plumley J., O’Quin J., Algeo T.P., Rupprecht C.E., Slate D. (2013). Costs of Raccoon Rabies Incidents in Cattle Herds in Hampshire County, West Virginia, and Guernsey County, Ohio. J. Am. Vet. Med. Assoc..

[53] Curk A. (1990). Economic losses due to rabies in Slovenia. Zb. Vet. Fak. Univerza Ljubl..

[54] Cusick M., Humphrey G. (1981). The Cost of One Rabid Dog--California. MMWR. Morb. Mortal. Wkly. Rep..

[55] Diamante E.O., Herrada N.J., Lachica Z.P.T., Oguis G.F.R., Alviola P.A., Mata M.A.E. (2021). Cost Optimization of the Intensified Rabies Control Program in Davao City, Philippines Using Linear Programming. Philipp. J. Sci..

[56] Diego A.I.d., Valotta J.R. (1973). Bat-transmitted rabies. Situation in Argentina. Revista de Medicina Veterinaria, Argentina.

[57] Dufour B., Aubert M., Bonnel A., Toma B. (1989). Control of rabies in cattle in 1987: Costs and benefits. Point Vétérinaire.

[58] Durr S., Mindekem R., Kaninga Y., Doumagoum Moto D., Meltzer M.I., Vounatsou P., Zinsstag J. (2009). Effectiveness of Dog Rabies Vaccination Programmes: Comparison of Owner-Charged and Free Vaccination Campaigns. Epidemiol. Infect..

[59] Dutta J.K. (1996). Rabies Prevention: Cost to an Indian Laborer. JAMA.

[60] Elser J.L., Bigler L.L., Anderson A.M., Maki J.L., Lein D.H., Shwiff S.A. (2016). The Economics of a Successful Raccoon Rabies Elimination Program on Long Island, New York. PLoS Neglected Trop. Dis..

[61] Elser J.L., Hatch B.G., Taylor L.H., Nel L.H., Shwiff S.A. (2018). Towards Canine Rabies Elimination: Economic Comparisons of Three Project Sites. Transbound. Emerg. Dis..

[62] Ferdous J., Islam A., Machalaba C., Feferholtz Y., Rahman M.A., Hagan E., Berthe F.C., Daszak P., Karesh W.B., Flora M.S. (2020). Economic Burden of Rabies and Its Impact in Bangladesh through a One Health Approach. Int. J. Infect. Dis..

[63] Fishbein D.B., Miranda N.J., Merrill P., Camba R.A., Meltzer M., Carlos E.T., Bautista C.F., Sopungco P.V., Mangahas L.C., Hernandez L.M. (1991). Rabies Control in the Republic of the Philippines: Benefits and Costs of Elimination. Vaccine.

[64] Fitzpatrick M.C., Hampson K., Cleaveland S., Mzimbiri I., Lankester F., Lembo T., Meyers L.A., Paltiel A.D., Galvani A.P. (2014). Cost-Effectiveness of Canine Vaccination to Prevent Human Rabies in Rural Tanzania. Ann. Intern. Med..

[65] Fitzpatrick M.C., Shah H.A., Pandey A., Bilinski A.M., Kakkar M., Clark A.D., Townsend J.P., Abbas S.S., Galvani A.P. (2016). One Health Approach to Cost-Effective Rabies Control in India. Proc. Natl. Acad. Sci. United States Am..

[66] Foroutan P., Meltzer M.I., Smith K.A. (2002). Cost of Distributing Oral Raccoon-Variant Rabies Vaccine in Ohio: 1997–2000. J. Am. Vet. Med. Assoc..

[67] Foroutan P. (2003). Agent-Based Modeling of Raccoon Rabies Epidemic and Its Economic Consequences.

[68] Freuling C., Seihorst T., Bätza H.J., Müller T. (2008). The Financial Challenge of Keeping a Large Region Rabies-Free—The EU Example. Dev. Biol..

[69] Gharpure R., Mitchell K.C., Dolan S., Iverson S.A., Feldman K.A. (2019). Low-Cost Animal Rabies Vaccination Clinics in Maryland Facilitate Access to Rabies Vaccination for Pets. Vector-Borne Zoonotic Dis..

[70] González-Roldán J.F., Undurraga E.A., Meltzer M.I., Atkins C., Vargas-Pino F., Gutiérrez-Cedillo V., Hernández-Pérez J.R. (2021). Cost-Effectiveness of the National Dog Rabies Prevention and Control Program in Mexico, 1990–2015. PLoS Neglected Trop. Dis..

[71] Göpfertová D., Walter G. (1983). Economic cost and losses in anti-rabies prophylaxis. Ceskoslov. Epidemiol. Mikrobiol. Immunol..

[72] Gordon E.R., Krebs J.W., Rupprecht C.R., Real L.A., Childs J.E. (2005). Persistence of Elevated Rabies Prevention Costs Following Post-Epizootic Declines in Rates of Rabies among Raccoons (Procyon Lotor). Prev. Vet. Med..

[73] Gulyukin A.M., Smolyaninov Y.I., Shabeykin A.A. (2016). The economic damage caused by rabies of agricultural animals in Russia. Russ. J. Agric. Socio-Econ. Sci..

[74] Hampson K., Ventura F., Steenson R., Mancy R., Trotter C., Cooper L., Abela-Ridder B., Knopf L., Ringenier M., Tenzin T. (2019). The Potential Effect of Improved Provision of Rabies Post-Exposure Prophylaxis in Gavi-Eligible Countries: A Modelling Study. Lancet Infect. Dis..

[75] Hardanahalli R.S., Annadani R.R., Undi M., Vijayashanakar V., Banerjee R., Mandya R.P. (2017). Economic Costs of Rabies Post Exposure Prophylaxis. Indian J. Community Health.

[76] Hatam N., Esmaelzade F., Mirahmadizadeh A.R., Keshavarz K., Rajabi A., Kazerooni P.A., Ataollahi M. (2014). Cost-Effectiveness of Rabies Post Exposure Prophylaxis in Iran. J. Res. Health Sci..

[77] Hatch B., Anderson A., Sambo M., Maziku M., Mchau G., Mbunda E., Mtema Z., Rupprecht C.E., Shwiff S.A., Nel L. (2017). Towards Canine Rabies Elimination in South-Eastern Tanzania: Assessment of Health Economic Data. Transbound. Emerg. Dis..

[78] Huot C., De Serres G., Duval B., Maranda-Aubut R., Ouakki M., Skowronski D.M. (2008). The Cost of Preventing Rabies at Any Cost: Post-Exposure Prophylaxis for Occult Bat Contact. Vaccine.

[79] Irmer S., Schlegel H.-L. (1981). Vaccination of young foxes as an alternative to rabies control. Cost benefit analysis. Berl. Und Münchener Tierärztliche Wochenschr..

[80] Jibat T.T.J., Mourits M.C.M., Hogeveen H. (2016). Incidence and Economic Impact of Rabies in the Cattle Population of Ethiopia. Prev. Vet. Med..

[81] Johnston D.H., Voigt D.R., MacInnes C.D., Bachmann P., Lawson K.F., Rupprecht C.E. (1988). An Aerial Baiting System for the Distribution of Attenuated or Recombinant Rabies Vaccines for Foxes, Raccoons, and Skunk. Rev. Infect. Dis..

[82] Kaare M., Lembo T., Hampson K., Ernest E., Estes A., Mentzel C., Cleaveland S. (2009). Rabies Control in Rural Africa: Evaluating Strategies for Effective Domestic Dog Vaccination. Vaccine.

[83] Kadowaki H., Duc P.P., Sato K., Phuong P.T.M., Hagiwara K., Makita K. (2018). Socio-Economic Factors Associated with Voluntary Rabies Control Measures in Vietnam. Prev. Vet. Med..

[84] Kahl W., Quander J., Posch J., Boegel K. (1978). Cost Analysis of Wildlife Rabies and Its Control in Europe. Zentralblatt Bakteriol. Parasitenkd. Infekt. Hyg. Erst Abt. Orig..

[85] Kallo V., Keita Z., Boka M., Tetchi M., Dagnogo K., Ouattara M., Amalaman D.M., Traore S., Gerber F., Lechenne M. (2022). Rabies Burden in Côte d’Ivoire. Acta Trop..

[86] Kayali U., Mindekem R., Hutton G., Ndoutamia A.G., Zinsstag J. (2006). Cost-Description of a Pilot Parenteral Vaccination Campaign against Rabies in Dogs in N’Djaména, Chad. Trop. Med. Int. Health.

[87] Keita Z., Gerber F., Lechenne M., Thiero O., Hattendorf J., Zinsstag J., Traoré A., Traoré A.K. (2020). Burden of Rabies in Mali. Acta Trop..

[88] Kemere P., Liddel M.K., Evangelou P., Slate D., Osmek S. (2000). Economic Analysis of a Large Scale Oral Vaccination Program to Control Raccoon Rabies.

[89] Khan M.H. (2002). The Economics of Prevention and Control of Rabies in Pakistan. J. Coll. Physicians Surg. Pak..

[90] Knobel D.L., Cleaveland S., Coleman P.G., Fèvre E.M., Meltzer M.I., Miranda M.E.G., Shaw A., Zinsstag J., Meslin F.X. (2005). Re-evaluating the burden of rabies in Africa and Asia. Bull. World Health Organ..

[91] Krebs J.W., Long-Marin S.C., Childs J.E. (1998). Causes, Costs, and Estimates of Rabies Postexposure Prophylaxis Treatments in the United States. J. Public Health Manag. Pract..

[92] Kreindel S.M., McGuill M., Meltzer M., Rupprecht C., DeMaria Jr. A. (1998). The Cost of Rabies Postexposure Prophylaxis: One State’s Experience. Public Health Rep..

[93] Kunkel A., Jeon S., Joseph H.C., Dilius P., Crowdis K., Meltzer M.I., Wallace R. (2021). The Urgency of Resuming Disrupted Dog Rabies Vaccination Campaigns: A Modeling and Cost-Effectiveness Analysis. Sci. Rep..

[94] Kwan N.C.L., Yamada A., Sugiura K. (2018). Benefit-Cost Analysis of the Policy of Mandatory Annual Rabies Vaccination of Domestic Dogs in Rabies-Free Japan. PLoS ONE.

[95] Larkins A.J., Reece J.F., Shaw A.P.M., Thrusfield M.V. (2020). An Economic Case Study of the Control of Dog-Mediated Rabies by an Animal Welfare Organisation in Jaipur, India. Prev. Vet. Med..

[96] Lavan R.P., King A.I.M., Sutton D.J., Tunceli K. (2017). Rationale and Support for a One Health Program for Canine Vaccination as the Most Cost-Effective Means of Controlling Zoonotic Rabies in Endemic Settings. Vaccine.

[97] Lis H. (1999). Epizootic situation after 6 years of vaccination of foxes against rabies in Poland. Med. Weter..

[98] Lushasi K., Brunker K., Rajeev M., Ferguson E.A., Jaswant G., Baker L., Biek R., Changalucha J., Cleaveland S., Czupryna A. (2023). Integrating Contact Tracing and Whole-Genome Sequencing to Track the Elimination of Dog-Mediated Rabies: An Observational and Genomic Study. eLife.

[99] Mann J.M., Burkhart M.J., Rollag O.J. (1980). Anti-Rabies Treatments in New Mexico: Impact of a Comprehensive Consultation-Biologics System. Am. J. Public Health.

[100] Mello A.K.M., Brumatti R.C., Neves D.A., Alcântara L.O.B., Araújo F.S., Gaspar A.O., Lemos R.A.A. (2019). Bovine Rabies: Economic Loss and Its Mitigation through Antirabies Vaccination. Pesqui. Vet. Bras..

[101] Meltzer M.I. (1996). Assessing the Costs and Benefits of an Oral Vaccine for Raccoon Rabies: A Possible Model. Emerg. Infect. Dis..

[102] Meltzer M.I., Rupprecht C.E. (1998). A Review of the Economics of the Prevention and Control of Rabies: Part 1: Global Impact and Rabies in Humans. Pharmacoeconomics.

[103] Mindekem R., Lechenne M.S., Naissengar K.S., Oussiguéré A., Kebkiba B., Moto D.D., Alfaroukh I.O., Ouedraogo L.T., Salifou S., Zinsstag J. (2017). Cost Description and Comparative Cost Efficiency of Post-Exposure Prophylaxis and Canine Mass Vaccination against Rabies in N’Djamena, Chad. Front. Vet. Sci..

[104] Miranda L.M., Miranda M.E., Hatch B., Deray R., Shwiff S., Roces M.C., Rupprecht C.E. (2017). Towards Canine Rabies Elimination in Cebu, Philippines: Assessment of Health Economic Data. Transbound. Emerg. Dis..

[105] Miranda M.E., Miranda N.L.J. (1997). City canine rabies elimination campaign: Manpower and program costs. Épidémiologie Santé Anim..

[106] Monroe B., Ludder F., Dilius P., Crowdis K., Lohr F., Cleaton J., Gamble L., Blanton J., Etheart M., Pieracci E.G. (2021). Every Dog Has Its Data: Evaluation of a Technology-Aided Canine Rabies Vaccination Campaign to Implement a Microplanning Approach. Front. Public Health.

[107] Motschwiller E. (1988). Epidemiological and Economic Aspects of Canine Rabies and its Control in Third World Countries. Ph.D. Thesis.

[108] Murphy J., Sifri C.D., Pruitt R., Hornberger M., Bonds D., Blanton J., Ellison J., Cagnina R.E., Enfield K.B., Shiferaw M. (2019). Human Rabies–Virginia, 2017. MMWR. Morb. Mortal. Wkly. Rep..

[109] Nguyen H.T., Le N.D., Pham T.N., Urabe M.I., Afriyie D.O., Otsu S., Tran D.N., Tran H.G., Nguyen H.V., Le H.T. (2019). Evaluation of Vietnam’s Post-Exposure Prophylaxis Delivery System, 2017. Vaccine.

[110] Manna N., Das S., Rahaman S.K.S. (2022). Debasis Das Economic Burden, Knowledge, and Practice Related to Prevention of Rabies: A Cross-Sectional Study among Animal Bite Victims Attending Rabies Immunization Clinic, Medical College and Hospital, Kolkata. Natl. J. Physiol. Pharm. Pharmacol..

[111] Nujum Z.T., Asaria M., Kurup K.K., Mini M., Mazumdar S., Daptardar M., Tiwari H. (2024). Cost-Effectiveness of One Health Interventions for Rabies Elimination: A Systematic Review. Trans. R. Soc. Trop. Med. Hyg..

[112] Oļševskis E., Lamberga K., Liepiņš E. (2012). Cost Efficiency of Rabies Oral Vaccination Strategies Implemented in Latvia from 1991 to 2011.

[113] Pieracci E.G. (2019). Vital Signs: Trends in Human Rabies Deaths and Exposures—United States, 1938–2018. Morb. Mortal. Wkly. Rep..

[114] Pinto H. (2011). de B.F.; Assis, A.; Pinto, R.M.; Monteiro, S.L.P.; Pinheiro, S.R. Cost benefit analysis of the activities for human rabies prevention and the activities for canine rabies control in Mogi-Guaçu municipality, state of Sao Paulo, from 2000 to 2004. Veterinária Zootec..

[115] Rahmanian V., Shakeri H., Jahromi A.S., Shakeri M., Khoubfekr H., Hatami I. (2020). Epidemiological Characteristic of Animal Bite and Direct Economic Burden of Rabies Vaccination in the Southern of Iran. Am. J. Anim. Vet. Sci..

[116] Recuenco S., Cherry B., Eidson M. (2007). Potential Cost Savings with Terrestrial Rabies Control. BMC Public Health.

[117] Recuenco S., Eidson M., Cherry B., Johnson G. (2009). Risk-Based Cost Modelling of Oral Rabies Vaccine Interventions for Raccoon Rabies. Zoonoses Public Health.

[118] Ribadeau Dumas F., N’Diaye D.S., Paireau J., Gautret P., Bourhy H., Le Pen C., Yazdanpanah Y. (2015). Cost-Effectiveness of Rabies Post-Exposure Prophylaxis in the Context of Very Low Rabies Risk: A Decision-Tree Model Based on the Experience of France. Vaccine.

[119] Rimhanen-Finne R., Ollgren J., Gadd T., Nokireki T. (2023). Notifications of Suspected Rabies Exposure Increased in Finland: 26 Years of One Health Surveillance, 1995–2020. Infect. Dis..

[120] Rosatte R., Donovan D., Allan M., Howes L.-A., Silver A., Bennett K., MacInnes C., Davies C., Wandeler A., Radford B. (2001). Emergency Response to Raccoon Rabies Introduction into Ontario. J. Wildl. Dis..

[121] Sambo M., Johnson P.C.D., Hotopp K., Changalucha J., Cleaveland S., Kazwala R., Lembo T., Lugelo A., Lushasi K., Maziku M. (2017). Comparing Methods of Assessing Dog Rabies Vaccination Coverage in Rural and Urban Communities in Tanzania. Front. Vet. Sci..

[122] Sartore S., Mulatti P., Trestini S., Lorenzetto M., Gagliazzo L., Marangon S., Bonfanti L. (2018). The Economic Implications of Sylvatic Rabies Eradication in Italy. Zoonoses Public Health.

[123] Sasaki D.M., Gooch J.M. (1983). Cost Effectiveness of Hawaii’s Anti-Rabies Quarantine Program. Hawaii Med. J..

[124] Schrodt C.A., Dilius P., Gibson A.D., Crowdis K., Fénelon N., Ross Y., Bonaparte S., Joseph H.C., Wallace R.M. (2023). Electronic Application for Rabies Management Improves Surveillance, Data Quality, and Investigator Experience in Haiti. Front. Vet. Sci..

[125] Scott T.P., Coetzer A., Nel L.H. (2016). Rabies in Namibia, More than a Horrendous Disease: The Social, Environmental and Economic Challenges Faced. Handbook on Africa: Challenges and Issues of the 21st Century.

[126] Selhorst T., Schlüter H. (1997). Cost-benefit analysis of the oral immunization strategy for the control of rabies in fox populations. Épidémiologie Santé Anim..

[127] Selhorst T., Thulke H.H., Müller T., Thrusfield M.V., Goodall E.A. (2000). Threshold Analysis of Cost-Efficient Oral Vaccination Strategies against Rabies in Fox (Vulpes Vulpes) Populations.

[128] Selhorst T., Thulke H.-H., Müller T. (2001). Cost-Efficient Vaccination of Foxes (Vulpes Vulpes) against Rabies and the Need for a New Baiting Strategy. Prev. Vet. Med..

[129] Shwiff S.A., Brown V.R., Dao T.T., Elser J., Trung H.X., Tien N.N., Huong N.T., Huong N.T.T., Riewpaiboon A., Ernst K. (2018). Estimating the Economic Impact of Canine Rabies to Viet Nam 2005-2014. PLoS Neglected Trop. Dis..

[130] Shwiff S., Aenishaenslin C., Ludwig A., Berthiaume P., Bigras-Poulin M., Kirkpatrick K., Lambert L., Bélanger D. (2013). Bioeconomic Modelling of Raccoon Rabies Spread Management Impacts in Quebec, Canada. Transbound. Emerg. Dis..

[131] Shwiff S.A., Elser J.L., Ernst K.H., Shwiff S.S., Anderson A.M. (2018). Cost-Benefit Analysis of Controlling Rabies: Placing Economics at the Heart of Rabies Control to Focus Political Will. Rev. Sci. Tech. (Int. Off. Epizoot.).

[132] Shwiff S.A., Hatch B., Anderson A., Nel L.H., Leroux K., Stewart D., de Scally M., Govender P., Rupprecht C.E. (2016). Towards Canine Rabies Elimination in KwaZulu-Natal, South Africa: Assessment of Health Economic Data. Transbound. Emerg. Dis..

[133] Shwiff S.A., Kirkpatrick K.N., Sterner R.T. (2008). Economic Evaluation of an Oral Rabies Vaccination Program for Control of a Domestic Dog-Coyote Rabies Epizootic: 1995-2006. J. Am. Vet. Med. Assoc..

[134] Shwiff S.A., Nunan C.P., Kirkpatrick K.N., Shwiff S.S. (2011). A Retrospective Economic Analysis of the Ontario Red Fox Oral Rabies Vaccination Programme. Zoonoses Public Health.

[135] Shwiff S.A., Sterner R.T., Hale R., Jay M.T., Sun B., Slate D. (2009). Benefit Cost Scenarios of Potential Oral Rabies Vaccination for Skunks in California. J. Wildl. Dis..

[136] Shwiff S.A., Sterner R.T., Jay M.T., Parikh S., Bellomy A., Meltzer M.I., Rupprecht C.E., Slate D. (2007). Direct and Indirect Costs of Rabies Exposure: A Retrospective Study in Southern California (1998-2002). J. Wildl. Dis..

[137] Shwiff S.A., Sweeney S.J., Elser J.L., Miller R.S., Farnsworth M.L., Nol P., Shwiff S.S., Anderson A.M. (2016). A Benefit-Cost Analysis Decision Framework for Mitigation of Disease Transmission at the Wildlife–Livestock Interface. Hum.-Wildl. Interact..

[138] Slate D., Kirby J.D., Morgan D.P., Algeo T.P., Trimarchi C.V., Nelson K.M., Rudd R.J., Randall A.R., Carrara M.S., Chipman R.B. (2017). Cost and Relative Value of Road Kill Surveys for Enhanced Rabies Surveillance in Raccoon Rabies Management. Trop. Med. Infect. Dis..

[139] Sparkes J., Ballard G., Fleming P.J.S., Brown W. (2017). Social, Conservation and Economic Implications of Rabies in Australia. Aust. Zool..

[140] Sreenivasan N., Li A., Shiferaw M., Tran C.H., Wallace R., Blanton J., Knopf L., Abela-Ridder B., Hyde T., Siddiqi U.R. (2019). Overview of Rabies Post-Exposure Prophylaxis Access, Procurement and Distribution in Selected Countries in Asia and Africa, 2017–2018. Vaccine.

[141] Sterner R.T., Kling M.A., Shwiff S.A., Slate D. (2003). Oral Rabies Vaccination: Reducing Economic Uncertainty via Response Surface Analysis.

[142] Sterner R.T. Economic Modeling of Oral Rabies Vaccination: Issues and Concepts. Proceedings of the Vertebrate Pest Conference.

[143] Sterner R.T., Meltzer M.I., Shwiff S.A., Slate D. (2009). Tactics and Economics of Wildlife Oral Rabies Vaccination, Canada and the United States. Emerg Infect Dis.

[144] Sterner R.T., Smith G.C. (2006). Modelling Wildlife Rabies: Transmission, Economics, and Conservation. Biol. Conserv..

[145] Sterner R.T., Sun B. Relative Factor Costs of Wildlife Rabies Impacts in the U.S.. Proceedings of the Vertebrate Pest Conference.

[146] Taylor E., Prada J.M., Del Rio Vilas V., Undurraga E.A., Wallace R., Horton D.L. (2023). Cost-Effectiveness Analysis of Integrated Bite Case Management and Sustained Dog Vaccination for Rabies Control. Am. J. Trop. Med. Hyg..

[147] Tenzin, Ward M.P., Wangdi K. (2012). Human and Animal Rabies Prevention and Control Cost in Bhutan, 2001-2008: The Cost-Benefit of Dog Rabies Elimination. Vaccine.

[148] Țibru I., Chirodea C., Gașpar C. (2018). The Economic Impact of Rabies in the Endemic Zones. Lucr. Stiintifice—Univ. De Stiinte Agric. A Banat. Timis. Med. Vet..

[149] Uhaa I.J., Dato V.M., Sorhage F.E., Beckley J.W., Roscoe D.E., Gorsky R.D., Fishbein D.B. (1992). Benefits and Costs of Using an Orally Absorbed Vaccine to Control Rabies in Raccoons. J. Am. Vet. Med. Assoc..

[150] Undurraga E.A., Meltzer M.I., Tran C.H., Atkins C.Y., Etheart M.D., Millien M.F., Adrien P., Wallace R.M. (2017). Cost-Effectiveness Evaluation of a Novel Integrated Bite Case Management Program for the Control of Human Rabies, Haiti 2014-2015. Am. J. Trop. Med. Hyg..

[151] Undurraga E.A., Millien M.F., Allel K., Etheart M.D., Cleaton J., Ross Y., Crowdis K., Medley A., Vos A., Maciel E. (2020). Costs and Effectiveness of Alternative Dog Vaccination Strategies to Improve Dog Population Coverage in Rural and Urban Settings during a Rabies Outbreak. Vaccine.

[152] Valenzuela L.M., Jayme S.I., Amparo A.C.B., Taylor L.H., Cruz M.Z.D., Licuan D.A., Gamal-Bitao R., Nel L.H. (2017). The Ilocos Norte Communities against Rabies Exposure Elimination Project in the Philippines: Epidemiological and Economic Aspects. Front. Vet. Sci..

[153] Vos A., Ün H., Hampson K., De Balogh K., Aylan O., Freuling C.M., Müller T., Fooks A.R., Johnson N. (2014). Bovine Rabies in Turkey: Patterns of Infection and Implications for Costs and Control. Epidemiol. Infect..

[154] Wang D.L., Zhang X.F., Wang X.C., Wang Y.T., Zhang R., Chen Y.Y., Wang Q., Yue N., Bao C.C., Zhou M.H. (2019). Cost-effectiveness analysis of rabies immunization strategy based on dynamic-decision tree model. Zhonghua Yu Fang Yi Xue Za Zhi.

[155] Wang W., Zhang R., Lin J., Sun J., Lyu H. (2017). Economic burden of post-exposure of rabies in Zhejiang province. Chin. J. Vector Biol. Control..

[156] Wera E., Mourits M.C.M., Hogeveen H. (2017). Cost-Effectiveness of Mass Dog Rabies Vaccination Strategies to Reduce Human Health Burden in Flores Island, Indonesia. Vaccine.

[157] Wera E., Mourits M.C.M., Siko M.M., Hogeveen H. (2017). Cost-Effectiveness of Mass Dog Vaccination Campaigns against Rabies in Flores Island, Indonesia. Transbound. Emerg. Dis..

[158] Wera E., Velthuis A.G.J., Geong M., Hogeveen H. (2013). Costs of Rabies Control: An Economic Calculation Method Applied to Flores Island. PLoS ONE.

[159] Whitehouse E.R., Person M.K., Brown C.M., Slavinski S., Rao A.K., Blanton J.D. (2021). Evaluating Surveillance for and Estimating Administration of Rabies Postexposure Prophylaxis in the United States, 2012–2018. PLoS Neglected Trop. Dis..

[160] Woodruff B.A., Jones J.L., Eng T.R. (1991). Human Exposure to Rabies from Pet Wild Raccoons in South Carolina and West Virginia, 1987 through 1988. Am. J. Public Health.

[161] World Health Organization (1989). FOX RABIES: Prophylaxis of Fox Rabies: A Cost-Benefit Study. Wkly. Epidemiol. Rec..

[162] Youssef S.B., Matter H.C., Schumacher C.L., Kharmachi H., Jemli J., Mrabet L., Gharbi M., Hammami S., Hicheri K.E., Aubert M.F.A. (1998). Field Evaluation of a Dog Owner, Participation-Based, Bait Delivery System for the Oral Immunization of Dogs against Rabies in Tunisia. Am. J. Trop. Med. Hyg..

[163] Zienius D., Bagdonas J., Dranseika A., Martinov S. (2002). Rabies Prevention Program in Lithuania during 1995-2000 Period. Biotechnol. Biotechnol. Equip..

[164] Zienius D., Petkevičius S., Vyšniauskas A. (2005). The 1995-2000 ORV Program in Lithuania Wildlife. Med. Weter..

[165] Zinsstag J., Dürr S., Penny M.A., Mindekem R., Roth F., Gonzalez S.M., Naissengar S., Hattendorf J. (2011). Transmission dynamics and cost-effectiveness of rabies control in dogs and humans in an African City. Rev. Médecine Trop..

[166] Zinsstag J., Lechenne M., Mindekem R., Naissengar S., Schelling E., Fooks A.R., Müller T. (2012). The Economics of Dog Rabies Control and the Potential for Combining It with Other Interventions.

